# Supplementary material for: Evolution in response to prophage activation attenuates the virulence of culturable Serratia symbiotica relatives of aphid endosymbionts
Source: mBio. 2025 Sep 8;16(10):e02041-25. doi: 10.1128/mbio.02041-25 (PMC12505973; doi:10.1128/mbio.02041-25)
Supplement: Fig. S1 — Growth rates and carrying capacities of evolved S. symbiotica clones cultured in vitro are uncorrelated with their virulence when injected into aphids. [file mbio.02041-25-s0002.pdf]

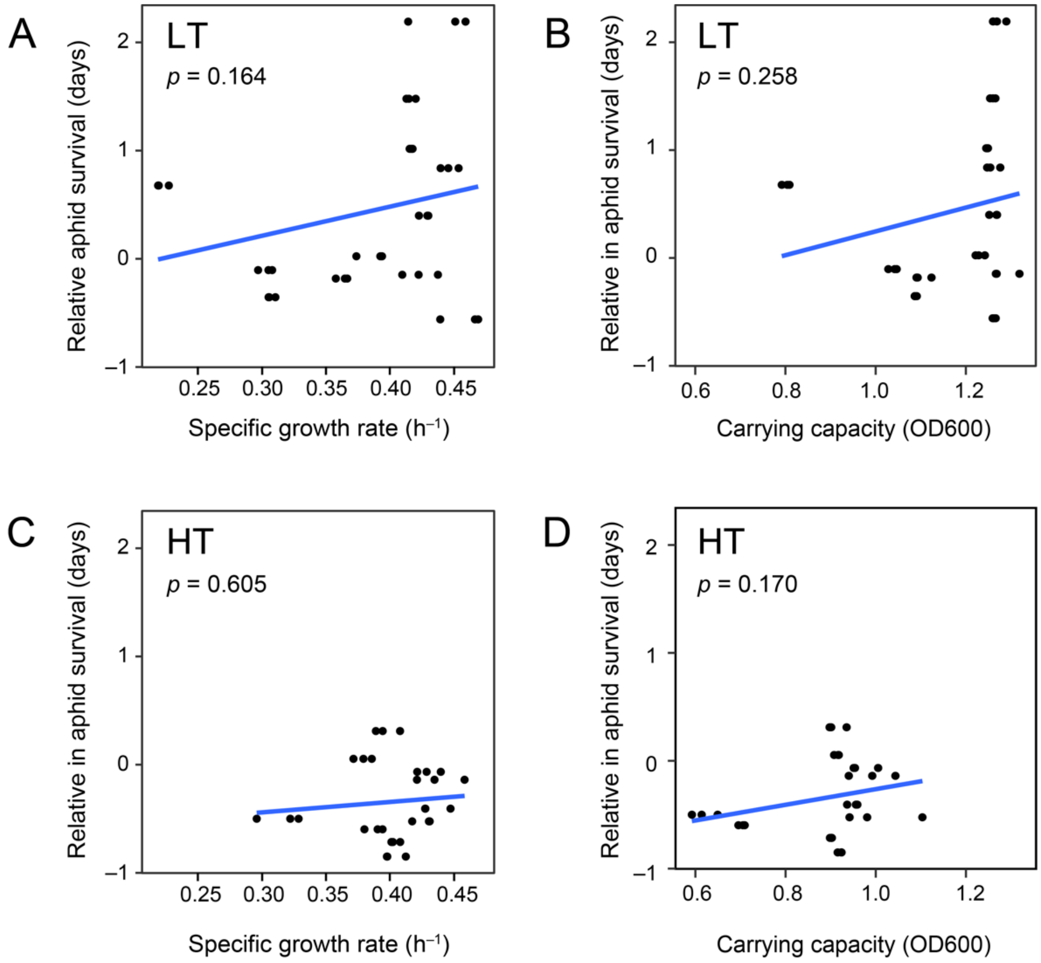

Fig. S1. Growth rates and carrying capacities of evolved *S. symbiotica* clones cultured *in vitro* are uncorrelated with their virulence when injected into aphids. Linear regressions were conducted between the average number of days aphids injected with the evolved clone survived relative to aphids injected with the ancestor strain (areas under the curves in **Fig. 5**) and parameters fit from each of three separate growth curve replicates for each clone. Each *p*-value is for rejecting the null hypothesis that there is no significant correlation (*t*-test that the slope of the fit line shown is not different from zero). (**A**) Correlation between growth rate and aphid survival for the LT-evolved clones. (**B**) Correlation between carrying capacity and aphid survival for the LT-evolved clones. (**C**) Correlation between growth rate and aphid survival for the HT-evolved clones. (**D**) Correlation between carrying capacity and aphid survival for the HT-evolved clones.
